# Supplementary material for: Type 2 diabetes and age-related cognitive decline over 40 years in Danish men–A cohort study based on the Danish Aging and Cognition (DanACo) cohort
Source: PLoS One. 2026 Jan 13;21(1):e0340622. doi: 10.1371/journal.pone.0340622 (PMC12798970; doi:10.1371/journal.pone.0340622)
Supplement: S1 Appendix — (DOCX) [file pone.0340622.s001.docx]

S1 Appendix 1. Supplementary Tables S1-S8

Table S1. Diagnostic codes (ICD-8 and ICD10) and the Anatomical Therapeutic Chemical (ATC)

codes used to identify men with type 1 diabetes, type 2 diabetes, and depression.

|  | **Type 1 diabetes** | **Type 2 diabetes** | **Depression** |
| --- | --- | --- | --- |
| **ICD-8** | 249 | 250 | 296.0, 296.2, 300.4 |
| **ICD-10** | E10 | E11, E13, E14, G590, G632,  H280, H360, M142, N083 | F32, F33 |
| **ATC-codes** | Insulin: A10A | Oral Antidiabetic Drugs (OAD): A10B  Insulin: A10A. | N06A |

Abbreviations: ICD: International Classification of Diseases; ATC: Anatomical Therapeutic Chemical.

The register-based measure of T2D was based on information from the Danish National Patient Register and the Danish National Prescription Register. Whereas the register-based measure of depression was based on information from the Danish Psychiatric Central Research register as well as the Prescription Register. The Danish National Patient register and the Danish Psychiatric Central Research register contain individual-level information on diagnosis and date of admission and discharge for all in-patient somatic and psychiatric hospital contacts, respectively. The psychiatric hospital contacts date back to 1969 whereas the somatic hospital contacts date back to 1977. From 1995 both registers also include outpatient contacts (Lynge, Sandegaard, & Rebolj, 2011; Mors, Perto, & Mortensen, 2011). Diagnoses have been coded according to the 8th Revision of the International Classification of Diseases (ICD-8) from 1969 through 1994, and the 10th Revision (ICD-10) from 1995 and onwards. The Danish National Prescription Register contains individual-level information on the date of prescription redemption, and the Anatomical Therapeutic Chemical (ATC) code for all prescription drugs sold in Danish community pharmacies from 1995 and onwards (Kildemoes, Sorensen, & Hallas, 2011).

Type 1 and type 2 diabetes were identified using the diagnostic and ATC-codes presented in Table S1. Men registered with both a type 1 and type 2 diabetes diagnosis using the above criteria were defined as having type 1 diabetes. Men who had redeemed a prescription with insulin were registered as having type 1 diabetes, unless they were also registered with a redemption of a prescription of an oral antidiabetic drug (OAD) or if the first prescription with insulin was redeemed after the age of 35.

Depression was identified using the diagnostic and ATC codes presented in Table S1.

Kildemoes, H. W., Sorensen, H. T., & Hallas, J. (2011). The Danish National Prescription Registry. *Scand J Public Health*, *39*(7 Suppl), 38–41. https://doi.org/10.1177/1403494810394717

Lynge, E., Sandegaard, J. L., & Rebolj, M. (2011). The Danish National Patient Register. *Scand J Public Health*, *39*(7 Suppl), 30–33. https://doi.org/10.1177/1403494811401482

Mors, O., Perto, G. P., & Mortensen, P. B. (2011). The Danish Psychiatric Central Research Register. *Scand J Public Health*, *39*(7 Suppl), 54–57. https://doi.org/10.1177/1403494810395825

Table S2. Number and proportion of men in the study population

having a self-reported and a register-based diagnosis of type 2 diabetes.

|  | **Register-based Type 2 diabetes** | | **Total** |
| --- | --- | --- | --- |
| **Self-reported Type 2 diabetes** | Yes | No |  |
| Yes | 685 | 76 | 761 |
| No | 47 | 4339 | 4386 |
| **Total** | 732 | 4415 | 5147 |

Table S3. Adjusted* associations between self-reported type 2 diabetes and IQ changes and having a significant decline in IQ stratified by baseline IQ in tertiles.

|  | **Lowest IQ tertile**  (n=1904) | | **Middle IQ tertile**  (n=1599) | | **Highest IQ tertile**  (n=1644) | |
| --- | --- | --- | --- | --- | --- | --- |
| **IQ changes** |  |  |  |  |  |  |
| Type 2 diabetes | *B* | (95% CI) | *B* | (95% CI) | *B* | (95% CI) |
| No | 0 | - | 0 | - | 0 | - |
| Yes | -1.71 | (-2.76,-0.66) | -1.32 | (-2.65,0.00) | -2.05 | (-3.50,-0.59) |
|  |  |  |  |  |  |  |
| **Significant decline in IQ** |  |  |  |  |  |  |
| Type 2 diabetes | OR | (95% CI) | OR | (95% CI) | OR | (95% CI) |
| No | 1 | - | 1 | - | 1 | - |
| Yes | 1.31 | (0.95,1.81) | 1.30 | (0.91,1.84) | 1.74 | (1.24,2.46) |

Abbreviations: IQ: Intelligence Quotient; *B*: unstandardized beta coefficients; CI: Confidence Interval; OR: Odds ratio.

*Adjusted for baseline IQ, age at follow-up, retest interval, years of education, smoking status, and depression.

^*^ *p* < 0.05, ^**^ *p* < 0.01, ^***^ *p* < 0.001.

Table S4. Adjusted* associations between self-reported type 2 diabetes and IQ changes and having a significant decline in IQ stratified by study origin

|  | LiKO-15  (n=2498) | | DiaKO-19  (n=2649) | |
| --- | --- | --- | --- | --- |
| **IQ changes** |  |  |  |  |
| Type 2 diabetes | *B* | (95% CI) | *B* | (95% CI) |
| No | 0 | - | 0 | - |
| Yes | -1.44 | (-2.68,-0.21) | -2.27 | (-3.13,-1.40) |
|  |  |  |  |  |
| **Significant decline in IQ** |  |  |  |  |
| Type 2 diabetes | OR | (95% CI) | OR | (95% CI) |
| No | 1 | - | 1 | - |
| Yes | 1.21 | (0.83,1.77) | 1.67 | (1.32,2.13) |

Abbreviations: LiKO-15: Lifestyle and Cognition Follow-up study 2015; DiaKO-19: Diabetes and Cognition Follow-up study 2019; IQ: Intelligence Quotient; *B*: unstandardized beta coefficients; CI: Confidence Interval; OR: Odds ratio.

*Adjusted for baseline IQ, age at follow-up, retest interval, years of education, smoking status, and depression.

^*^ *p* < 0.05, ^**^ *p* < 0.01, ^***^ *p* < 0.001.

Table S5: Post hoc analyses of the influence of the individual covariates of Model 2 in Table 3 on the association between self-reported type 2 diabetes and changes in IQ.

|  | **Crude** | | **Adjusted for**  **baseline IQ** | | **Adjusted for**  **education years** | | **Adjusted for**  **follow-up age** | | **Adjusted for**  **retest interval** | |
| --- | --- | --- | --- | --- | --- | --- | --- | --- | --- | --- |
|  | R^2^=0.003 | | R^2^=0.150 | | R^2^=0.021 | | R^2^=0.044 | | R^2^=0.014 | |
|  | *B* | [95% CI] | *B* | [95% CI] | *B* | [95% CI] | *B* | [95% CI] | *B* | [95% CI] |
| Type 2 diabetes | -1.59 | [-2.33,-0.84] | -2.77 | [-3.46,-2.08] | -2.07 | [-2.81,-1.33] | -0.87 | [-1.61,-0.14] | -1.16 | [-1.91,-0.41] |
| Baseline IQ |  |  | -0.25 | [-0.26,-0.23] |  |  |  |  |  |  |
| Age at follow-up |  |  |  |  | -0.52 | [-0.62,-0.41] |  |  |  |  |
| Retest interval |  |  |  |  |  |  | -0.47 | [-0.53,-0.40] |  |  |
| Years of education |  |  |  |  |  |  |  |  | -0.24 | [-0.30,-0.18] |

Abbreviations: IQ: Intelligence Quotient; *B*: unstandardized beta coefficients; CI: Confidence Interval.

^*^ *p* < 0.05, ^**^ *p* < 0.01, ^***^ *p* < 0.001.

Table 3. Association between self-reported type 2 diabetes and IQ changes and having a significant decline in IQ in Danish men (n=5,147) in unadjusted and adjusted linear- and logistic regression analyses. Model 2 adjusted for register-based depression.

|  | Model 2 | |
| --- | --- | --- |
| **IQ changes** |  |  |
| T2D | *B* | (95% CI) |
| No | 0 | - |
| Yes | -1.75*** | (-2.42,-1.07) |
| **Significant decline in IQ** |  |  |
| T2D | OR | (95% CI) |
| No | 1 | - |
| Yes | 1.40*** | (1.16,1.70) |

Abbreviations: IQ: Intelligence Quotient; T2D: Type 2 diabetes; *B*: unstandardized beta coefficients; CI: Confidence Interval; OR: Odds ratio.

Adjusted for baseline IQ, age at follow-up, retest interval, years of education, smoking status, and register-based depression.

^*^ *p* < 0.05, ^**^ *p* < 0.01, ^***^ *p* < 0.001.

Table S7. Characteristics of non-participants and participants presented according to register-based type 2 diabetes status.

|  | Non-participants | | |  | Participants | | |  |
| --- | --- | --- | --- | --- | --- | --- | --- | --- |
|  | Total | Type 2 diabetes | | p-value | Total | Type 2 diabetes | | p-value |
| Number, N | (n=32104) | No (n=26693) | Yes (n=5411) |  | (n=5147) | No (n=4415) | Yes (n=732) |  |
| Birth year, Mean (SD) | 1954.6 (3.2) | 1954.8 (3.2) | 1953.9 (3.1) | <0.001 | 1954.2 (3.2) | 1954.3 (3.2) | 1953.5 (3.0) | <0.001 |
| Age at invitation to follow-up | 63.5 (4.2) | 63.2 (4.3) | 64.6 (3.8) | <0.001 | 64.1 (4.2) | 63.9 (4.2) | 65.4 (3.5) | <0.001 |
| *Variables from the conscript board examination* | | | | | | | | |
| *Age, Mean (SD) | 20.0 (1.8) | 20.0 (1.8) | 19.8 (1.7) | <0.001 | 20.4 (2.1) | 20.4 (2.1) | 20.2 (2.0) | 0.002 |
| *IQ, Mean (SD) | 89.3 (17.8) | 90.0 (17.6) | 85.4 (18.1) | <0.001 | 100.0 (15.0) | 100.7 (14.7) | 95.7 (16.2) | <0.001 |
| *Educational level |  |  |  | <0.001 |  |  |  | <0.001 |
| Low | 15965 (49.8) | 12722 (47.7) | 3243 (60.0) |  | 1433 (27.9) | 1138 (25.8) | 295 (40.5) |  |
| Medium | 9118 (28.4) | 7762 (29.1) | 1356 (25.1) |  | 1715 (33.4) | 1474 (33.4) | 241 (33.1) |  |
| High | 6976 (21.8) | 6170 (23.1) | 806 (14.9) |  | 1989 (38.7) | 1796 (40.7) | 193 (26.5) |  |
| *Height in cm, Mean (SD) | 178.5 (6.6) | 178.7 (6.6) | 177.8 (6.7) | <0.001 | 179.5 (6.5) | 179.7 (6.4) | 178.5 (6.8) | <0.001 |
| *Weight in kg, Mean (SD) | 69.0 (9.9) | 68.3 (9.2) | 72.5 (12.0) | <0.001 | 69.6 (9.1) | 69.1 (8.5) | 72.6 (11.9) | <0.001 |
| *BMI, N (%) |  |  |  | <0.001 |  |  |  | <0.001 |
| <18.5 | 2611 (8.5) | 2321 (9.1) | 290 (5.6) |  | 340 (7.0) | 303 (7.3) | 37 (5.4) |  |
| 18.5-25 | 25201 (81.8) | 21458 (83.7) | 3743 (72.1) |  | 4087 (84.7) | 3579 (86.5) | 508 (74.1) |  |
| 25-30 | 2627 (8.5) | 1674 (6.5) | 953 (18.4) |  | 365 (7.6) | 243 (5.9) | 122 (17.8) |  |
| >=30 | 385 (1.2) | 183 (0.7) | 202 (3.9) |  | 33 (0.7) | 14 (0.3) | 19 (2.8) |  |

Abbreviations: SD: Standard Deviation; IQ: Intelligence Quotient; BMI: Body Mass Index.

*Missing information. Non-participants, n missing: Age: 11, IQ: 1, Educational level: 45, Height: 89, Weight: 1269, BMI: 1280; Participants, n missing: Educational level: 10, Height: 13, Weight: 322, BMI: 322

Table S8. Associations between self-reported type 2 diabetes and IQ changes and having a significant decline in IQ in Danish men (n=5,147) in unadjusted and adjusted linear- and logistic regression analyses applying inverse probability weights to account for selection bias.

|  | **Main analyses** | | | | | | **Sensitivity analyses** | |
| --- | --- | --- | --- | --- | --- | --- | --- | --- |
|  | Crude | | Model 1 | | Model 2 | | Model 3† | |
| **IQ changes** |  | |  | |  | |  | |
| Type 2 diabetes | *B* | (95% CI) | *B* | (95% CI) | *B* | (95% CI) | *B* | (95% CI) |
| No | 0 | - | 0 | - | 0 | - | 0 | - |
| Yes | -1.95*** | (-2.87,-1.03) | -2.33*** | (-3.23,-1.43) | -2.33*** | (-3.22,-1.43) | -2.32*** | (-3.26,-1.38) |
|  |  |  |  |  |  |  |  |  |
| **Significant decline in IQ** |  |  |  |  |  |  |  |  |
| Type 2 diabetes | OR | (95% CI) | OR | (95% CI) | OR | (95% CI) | OR | (95% CI) |
| No | 1 | - | 1 | - | 1 | - | 1 | - |
| Yes | 1.32** | (1.08,1.62) | 1.36** | (1.09,1.68) | 1.34** | (1.08,1.67) | 1.37** | (1.09,1.72) |

Abbreviations: IQ: Intelligence Quotient; *B*: unstandardized beta coefficients; CI: Confidence Interval; OR: Odds ratio.

Model 1: adjusted for baseline IQ, age at follow-up, retest interval, and years of education.

Model 2: adjusted for model 1 + smoking status, and depression.

Model 3: adjusted for model 2 + baseline BMI.

^*^ *p* < 0.05, ^**^ *p* < 0.01, ^***^ *p* < 0.001.

†Due to missing information on BMI, model 3 only includes 4825 observations.

Increment in R²: Model 1 = 0.66%, Model 2 = 0.65%, Model 3 = 0.64%.
