# Supplementary material for: Type 2 diabetes and age-related cognitive decline over 40 years in Danish men–A cohort study based on the Danish Aging and Cognition (DanACo) cohort
Source: PLoS One. 2026 Jan 13;21(1):e0340622. doi: 10.1371/journal.pone.0340622 (PMC12798970; doi:10.1371/journal.pone.0340622)
Supplement: Attachment — Submitted filename: Response to reviewers.docx [file pone.0340622.s002.docx]

Response to reviewers

PONE-D-25-26526

Type 2 diabetes and age-related cognitive decline over 40 years in Danish men - A cohort study based on the Danish Aging and Cognition (DanACo) cohort

I want to sincerely thank both reviewers for taking the time to review the paper. I have responded to all comments below. Page and line numbers refer to the placement in the manuscript with track changes.

**Reviewers' comments:**

**Reviewer #1:** Dear Dr. Dr. Aleksandra Klisic,

Thank you for the opportunity to review the manuscript titled “Type 2 diabetes and age-related cognitive decline over 40 years in Danish men – A cohort study based on the Danish Aging and Cognition (DanACo) cohort,” submitted to PLOS ONE.

This manuscript presents a well-designed and methodologically robust investigation into the long-term association between type 2 diabetes (T2D) and cognitive decline over a 40-year period, utilizing data from the Danish Aging and Cognition cohort. A notable strength of the study is the inclusion of pre-morbid cognitive ability assessed in young adulthood, which enhances the interpretability of the longitudinal findings and provides a key methodological advantage over many prior studies in the literature.

The authors report a modest yet statistically significant difference in IQ decline among men diagnosed with T2D compared to non-diabetic counterparts, alongside an increased risk of substantial cognitive decline as measured by the Reliable Change Index. The study’s methodological rigor is further supported by the use of both self-reported and registry-based diabetes diagnoses, as well as comprehensive adjustment for a range of relevant confounding variables, including depression, educational attainment, smoking status, and body mass index (BMI). These methodological strengths contribute to the overall robustness and credibility of the findings.

I find the manuscript to be a valuable and timely contribution to the expanding body of research on cognitive aging and metabolic
health. The study is methodologically sound and conceptually relevant. However, I recommend a few minor revisions aimed at enhancing the clarity of presentation and strengthening the contextual framing of the findings within the broader literature:

Response: Thank you for taking the time to carefully read and review our manuscript.

1. Clarify Clinical Significance: The observed IQ difference (~1.8 points) is statistically significant but may be perceived as small. While the authors note this in the discussion, a brief elaboration on the potential functional impact—or lack thereof—would help readers interpret the practical meaning of the result.

Response: We have added a sentence stating the lack of clinical relevance of a 1.81 IQ point difference: p. 20, lines 428-432.

*While a difference of 1.81 IQ points lacks clinical relevance and may not be noticeable for the individual, the difference in cognitive decline between men with and without T2D may increase with increasing age which is supported by the larger relative risk for dementia found with increasing age (29).*

2. Depression Interaction: The rationale for excluding depression as a moderating factor could be more clearly stated. A brief explanation in the discussion about why a synergistic effect between T2D and depression was not observed (e.g., cohort characteristics) would be welcome.

Response: We agree that the exploration of the hypothesized interaction between depression and T2D has not been presented sufficiently. We have thus decided to present the results from the stratified analyses in a new Table 2 and describe the results at the beginning of the results section. We have also elaborated the discussion of why we do not find a synergistic effect in the discussion section. P. 20 lines 434-441:

*Our a priori hypothesis of a synergistic effect of having both T2D and having had depression was not supported in the analysis. The reasons are not clear, but the all-male population may blunt the importance of depression. Co-morbid depression is more common in women with T2D (14), and our interaction analysis may not have had statistical power to evaluate effect modification by depression. Moreover, there could be a problem with misclassification, as unrecognized depression may be more common among men due to cultural norms and gender-related differences in symptom recognition and help-seeking behavior(30).*

3. Participation Bias: Given the relatively low participation rate (~14%), this limitation should be emphasized more clearly in both the abstract and conclusion sections to ensure appropriate interpretation of generalizability.

Response: We have stated the low participation rate in both abstract and conclusion. Furthermore,

following the suggestion of reviewer 2, we have attempted to account for potential selection bias by

applying inverse probability weights based on the predicted probability of participating in the

follow-up using baseline characteristics (IQ, education, and BMI) and age at invitation to the

follow-up examination. The analyses of Table 3 (originally tables 2 and 3) were rerun applying

these weights (Supplementary Table S8) and the estimated associations were comparable, thus

strengthening the validity or our conclusions and implying that the observed associations may

reflect those in the underlying source population.

4. Terminological Precision: The manuscript occasionally shifts between “IQ” and “cognitive ability.” Standardizing or clarifying the usage of these terms may improve precision for interdisciplinary readers.

Response: Thank you for making us aware of these shifts in terminology. We have adjusted the manuscript so that we use cognitive ability when we refer to the theoretical concept of intelligence, primarily in the Introduction and Discussion, and IQ when referring to the actual scores. We introduce IQ in the Methods section under the description of the two operationalization’s of cognitive decline and IQ is primarily used in the results section, but also when referring to specific estimates in the Abstract and Discussion.

5. Formatting and Flow: Some minor editorial polishing would improve clarity—especially in integrating Table 4 more directly into the narrative and improving typographic consistency.

Response: We have carefully edited the manuscript and the layout of the tables has been streamlined and unnecessary elements have been moved to the footnotes, to make the tables more easily readable.

I recommend that the manuscript be accepted pending minor revisions, as outlined above. This is a thoughtfully designed and well-contextualized study that offers a meaningful contribution to our understanding of the long-term cognitive consequences associated with type 2 diabetes.

**Reviewer #2:** Thank you for the opportunity to review this manuscript, which investigates the association between type 2 diabetes (T2D) and cognitive decline over 40 years in Danish men using the unique Danish Aging and Cognition (DanACo) cohort. The study leverages longitudinal cognitive data from young adulthood to late mid-life, aiming to address gaps in prior literature regarding pre-morbid cognitive assessment and long-term follow-up.

Response: Thank you for taking the time to carefully read and review our manuscript.

Title (Page 1, Lines 2–4)
The title specifies "Danish men" but does not clarify the male-only cohort in the introduction.

Response: It has now been specified in the aim that the study is based on a cohort of middle-aged Danish men. P. 5 lines 86-87:

*The primary aim of the present study was to investigate the influence of T2D on cognitive changes over an average of 44 years using a draft board intelligence test as baseline and a re-administration of the same intelligence test in a cohort of middle-aged Daish men.*

Abstract (Page 1, Lines 25–46; Page 9, Lines 24–46)
The abstract states the objective as investigating T2D’s influence on cognitive decline "over a period of more than 40 years" (Lines 26–28). However, the mean retest interval is 44 years (Page 15, Line 193), and the range (35.5–53.2 years, Page 11, Line 91) suggests significant variability.

Response: To emphasize the variability in retest interval length, we have rephrased it so that it now states: “over a period of on average 44 years”, and we have done so throughout the abstract and main text. However, we have decided to keep the term “over 40 years” in the title to keep the title short.

Results misalignment: The abstract reports a 1.81-IQ-point decline (Lines 38–39) but Table 2 (Page 18) cites 1.83 points.

Response: It has now been corrected so that all references to the main results of Table 3 (originally Table 2) throughout the abstract and main text is now from model 2 (1.81 IQ points).

Outcome ambiguity: "Significant IQ decline" is defined via the Reliable Change Index (RCI) (Lines 35–36), but the abstract omits the clinical relevance of the 13.2-IQ-point cutoff.

Response: We have specified the cut-off (cut-off: 13.2 IQ-points) in the abstract and have rephrased the conclusion to put more emphasis on the clinical relevance of the higher odds of having a significant IQ decline. P. 2 lines 47-48.

*Type 2 diabetes was associated with modestly greater cognitive decline and higher odds of a statistically significant (>13.2 IQ points) and clinically relevant decline.*

Introduction
Background/Rationale (Page 10, Lines 49–82)
The introduction hypothesizes synergistic effects of T2D and depression (Lines 81–82) but later dismisses this (Page 15, Lines 173–178) without reconciling the initial rationale.

Response: We agree that the exploration of the hypothesized interaction between depression and T2D has not been presented sufficiently. We have thus decided to present the results from the interaction analyses in a new Table 2 and describe the results at the beginning of the results section. We have also elaborated the discussion of why we do not find a synergistic effect in the discussion section. P. 20 lines 434-441:

*Our a priori hypothesis of a synergistic effect of having both T2D and having had depression was not supported in the analysis. The reasons are not clear, but the all-male population may blunt the importance of depression. Co-morbid depression is more common in women with T2D (14), and our interaction analysis may not have had the statistical power to detect an interaction between T2D and depression. Moreover, there could be a problem with misclassification, as unrecognized depression may be more common among men due to cultural norms and gender-related differences in symptom recognition and help-seeking behavior(30).*

The manuscript positions itself as unique in assessing pre-morbid cognition (Lines 62–68) but downplays the single prior study (Reference 12) addressing this (Lines 63–66).

Response: We have elaborated on the differences of the findings of our study and the study by Mottus et al in the Discussion, p. 19 lines 419-425:

*Consistent with the only prior study including a pre-morbid cognitive measure(12), we found that men with T2D had lower baseline IQ than those without T2D, indicating that lower cognitive ability may increase T2D risk. Unlike the prior study, however, the IQ difference widened at follow-up, and adjusting for baseline IQ strengthened the association, suggesting that T2D may accelerate cognitive decline and that the difference in late-life cognitive ability of men with and without T2D is not just a result of a lifelong lower ability among men with T2D.*

Objectives (Page 11, Lines 77–82)
The aim to analyze "potential synergistic effect" of T2D and depression (Line 82) is not evaluated in primary results (Table 2) or discussed robustly (Page 23, Lines 337–340).

Response: We agree that the results of the analyses exploring the interaction between depression and T2D have not been presented sufficiently. We have thus decided to present the results in a new Table 2 and describe the results in the beginning of the results section.

Methods
Study Design & Participants (Page 11–12, Lines 83–98)
157 participants lost due to "technical errors" (Page 11, Line 95) are excluded without detailing the nature of errors or potential bias.

Response: The data was lost due to a server malfunction at two separate occasions during the data collection. It was the military that handled the collection and extraction of data, and the server malfunctions resulted in all data collected during the two specific time periods (each covering around 2 weeks) were not saved to the server and could thus not be recovered. The timing of the errors were random and the participants chose the time of their examination themselves, and we did not invite the participants in any particular order based on their characteristics. We have added some more details in the methods section and added a sentence in the discussion about potential bias.

*Of the 5340 participants, all follow-up data for a total of 157 participants was lost due to server malfunction during two shorter time periods resulting in the deletion of data from all men participating during these periods. Page 5 lines 103-105:*

*Another limitation of the study was the loss of follow-up data for 157 participants due to server malfunctions during two shorter time periods. Since the participants chose their own examination times and were not invited in any specific order, the server malfunctions were not related to participant characteristics and could thus not result in selection bias. Page 22 lines 491-495.*

The 14.3% participation rate (Page 24, Line 368) and healthier profile of participants (Page 24, Lines 369–373) are noted but not quantitatively evaluated for impact on effect estimates.
Variables (Page 12–14, Lines 99–157)

Response: Thank you for this suggestion. We have now attempted to account for potential selection bias by applying inverse probability weights based on the predicted probability of participating in the follow-up using baseline characteristics (IQ, education, and BMI) and age at invitation to the follow-up examination. The analyses of Table 3 (originally tables 2 and 3) were rerun applying these weights and the estimated associations were comparable, thus strengthening the validity or our conclusions and implying that the observed associations may reflect those in the underlying source population.

Self-reported T2D classification uses inconsistent criteria between LiKO-15 and DiaKO-19 (Page 12, Lines 103–107). This heterogeneity is not addressed in sensitivity analyses.

Response: It is correct that the self-reported information on T2D was not consistent in LiKO-15 and DiaKO-19. However, we did use the same criteria for distinguishing between T1D and T2D for both cohorts (T1D if age of onset was before age 35 otherwise T2D). We also did an interaction analyses including an interaction term between T2D and study origin. There was no indication of interaction. The results of this analysis were not presented in the original submission, so we have now added them to the supplementary material (see Supplementary Table S4).

The BPP test switched from paper-and-pencil to computerized format between baseline and follow-up (Page 13, Line 130). Equivalence of these formats is asserted but not empirically validated.

Response: The change from paper-and-pencil to the computerized format was implemented in the autumn of 2010, and in a paper from 2021, I have together with colleagues, showed that the change coincided with a drop in mean IQ of 1.15 points. The mean was relatively stable from 2006 to 2010, dropped 1.5 IQ points and then remained relatively stable from 2011 to 2019. As the items have remained unchanged it suggests that the change of format is responsible for the drop in the mean number of items answered correctly. However, we have no reason to suspect that the change in format has introduced bias as we must expect that the change affects men with and without T2D equally. We have included a sentence on this matter in the Discussion, p. 23 lines 501-507:

*While the BPP-test items remained unchanged, the format was changed from paper-and-pencil at baseline to a computerized format at follow-up. In a study of all men examined by the conscription board from 2006 through 2019, it has been shown that the change in format coincided with a 1.5-point drop in mean IQ, suggesting that the format shift reduced scores slightly(30). However, we have no reason to suspect that the format change affected men with and without T2D differently, and therefore the change is unlikely to have introduced bias.*

The RCI formula uses a baseline SD of 9.5 and reliability (omega) of 0.82 (Page 13, Lines 140–142), but the source of these values is unreferenced.

Response: Thank you for pointing out the missing reference of the values. They are based on analyses of raw BPP scores, and are referenced in the cohort profile for the DanACo cohort (Grønkjær et al 2024). We have made this clear in the manuscript, p. 7 lines 151-152:

*(estimated using the baseline standard deviation of the BPP-score of 9.5 and the estimated test reliability (omega) of 0.82, see Grønkjær et al 2024(21)).*

Physical activity, diet, and cardiovascular comorbidities—established confounders in T2D-cognition pathways—are not adjusted for.

Response: Physical inactivity and diet could be potential confounders and we have included a sentence in the discussion highlighting the lack of data on these two factors. However, we have decided to not include cardiovascular co-morbidity as we consider this to be a mediator rather than a confounder. P. 23 lines 498-501:

*We had no information on physical inactivity and diet, which are both associated with T2D (31–33) and modest predictors of cognitive decline (34–36), and thus could have explained part of the association.*

Statistical Methods (Page 14–15, Lines 164–190)
The marginally significant T2D-depression interaction (p=0.046, Page 15, Line 177) is dismissed without sensitivity analysis.

Response: We are unaware of what types of sensitivity analyses the reviewer is asking for here.

Post hoc analyses reveal confounding patterns (Supplementary Table S3, Page 17, Lines 216–218), but the final model (Model 2) does not include retest interval, a key covariate.

Response: Thank you for making us aware of this mistake. Model 1 actually did include retest interval, but it was left out in the description in the main text and under the tables. We have now added retest interval in the description of model 1 in both the main text and under the tables.

Results
Participants & Descriptive Data (Page 15–16, Lines 192–208; Table 1)
Baseline BMI categories (Page 16, Lines 198–199) include "<18.5" (underweight), but this group’s relevance to T2D is unclear. No rationale for cutoff choices is provided.

Response: The chosen categorization is the standard categorization of BMI categories as defined by the WHO and the CDC and it is widely used in the literature on BMI. The association between underweight and T2D remains unclear with mixed findings in the extant literature. However, we have previously shown an inverse J-shaped association between BMI and BPP in Danish conscripts. Given that the size of the study population allowed for a more fine-grained categorization, we decided to give as much information as possible in Table 1.

Baseline BMI has 322 missing values (Page 16, Line 207), but analyses including BMI (Model 3) do not discuss implications of reduced sample size.

Response: The missing BMI values were caused by the fact that some conscription board districts did not record the weight. So, the missing registrations were not dependent on the weight of the conscripts, but was purely a matter of the district they were examined in. We have added a sentence on the implications of the reduced sample size in the discussion. P. 22 lines 495-498:

*Furthermore, 322 men had missing information on baseline BMI caused by some conscription board districts not registering weight at all. We thus consider the missing observations to be missing at random wherefore the estimates of the sensitivity analyses may be underestimated but not biased.*

Outcome Data & Main Results (Page 17–20; Tables 2–4)
The 1.81-IQ-point decline (Table 2) is termed "modest" (Page 20, Line 277), but the 26.7% vs. 20.5% rate of "significant decline" (Page 16, Line 197) is clinically meaningful yet underemphasized.

Response: We agree that the results on significant decline have been somewhat downplayed throughout the paper, and we have made an effort to highlight the substantially higher odds of having a significant decline among men with T2D compared to men without T2D.

For T2D duration ≥20 years (n=107, Page 19, Table 3), nonsignificant trends (p>0.05) are reported despite point estimates suggesting clinically relevant decline (e.g., OR=1.52 for significant decline).

Response: We are not completely sure what the reviewer is trying to convey with the comment. We believe that our description of the results on T2D duration from the analyses using self-reported T2D is correct (p. 15 lines 317-319). However, we find that the description of the results from the analyses using a register-based measure of T2D was somewhat downplayed. We have thus rephrased the description of results from Table 5 (originally 4) – in both the results section and in the beginning of the discussion:

Results p. 15, lines 319-322:

*but for men with register-based T2D there was a statistically significant larger decline and the odds of having a significant decline was doubled for men with a duration of 20 years or more compared to a duration of 0-9 years (Table 5).*

Discussion p. 17, lines 353-357:

*Moreover, there was a clear tendency of a larger decline with longer diabetes duration, and in the analysis using the register-based measure of T2D (Table 5) a doubling of the odds of having a significant decline was found for men with a duration of more than 20 years compared to men with a duration of 0-9 years*

Results for depression as an effect modifier (Page 15, Lines 170–178) are not quantified in tables.

Response: Thank you for this suggestion. We have decided to include the results from the interaction analyses in a new Table 2 in the beginning of the results section. We have also added tables showing the interaction analyses of baseline IQ and cohort study in the supplemental material.

Discussion
Key Findings & Interpretation (Page 20–23, Lines 275–348)
Mechanisms linking T2D to cognitive decline (Page 22, Lines 316–320) are discussed without direct evidence from the study.

Response: We agree that the paragraph on “Interpretation of findings” includes too many details with regard to the mechanisms linking T2D to cognitive decline, and the paragraph is not referencing our study sufficiently. We have deleted parts of the paragraph and have restructured and rewritten it to enhance the relation to our study.

Generalizability to women (Page 25, Lines 383–384) is claimed despite male-only data and acknowledged sex differences in T2D-depression comorbidity (Page 23, Line 339).

Response: In the discussion we state that our results might be generalizable to women based on the fact that previous studies of the association between T2D and cognitive decline have not reported sex differences in the association. We have rephrased our statement a bit to underline the speculative nature and we have also added a sentence on the possibility of different results regarding the potential interaction of T2D and depression. P. 23 lines 508-513:

*however, the two reviews of the association between T2D and cognitive decline (1,27) did not report any sex-differences suggesting that the modestly larger decline associated with T2D in the present study may potentially be generalizable to women. However, as co-morbid depression is more common in women with T2D it might have been possible to establish a clear interaction between T2D and depression among women.*

Limitations & Generalizability (Page 24–25, Lines 367–384)

The assertion that baseline characteristics were "similar" between participants and non-participants with T2D (Page 20, Lines 378–380) is unsupported by data (Supplementary Table S4 not shown).

Response: Table S8 (previously table S4) was part of the original supplementary material in the submission. So it is not clear why reviewer 2 writes that Table S4 was not shown. We do not write that the baseline characteristics were similar between participants and non-participants. What we do write is that the distribution of characteristics by T2D status was similar between participants and non-participants. However, given the addition of the extra analysis applying inverse probability weights we have deleted the statement from the discussion, as it is no longer relevant.

The healthier, more educated cohort (Page 24, Lines 373–374) may attenuate observed effects, but the magnitude of this bias is not estimated.

Response: Again, thank you for suggesting quantitative bias analysis. We have now attempted to account for potential selection bias by applying inverse probability weights based on the predicted probability of participating in the follow-up using baseline characteristics (IQ, education, and BMI) and age at invitation to the follow-up examination. The analyses of Table 3 (originally tables 2 and 3) were rerun applying these weights and the estimated associations were comparable, thus strengthening the validity or our conclusions and implying that the observed associations may reflect those in the underlying source population.
